# Study Protocol: The influence of Running Therapy on executive functions and sleep of prisoners

**DOI:** 10.12688/f1000research.6469.1

**Published:** 2015-06-15

**Authors:** Jesse Meijers, Joke Harte, Gerben Meynen, Pim Cuijpers

**Affiliations:** 1 Department of Clinical Neuropsychology, Faculty of Psychology and Education, VU University Amsterdam, Amsterdam, 1081BT, Netherlands; 2 Department of Criminal Law and Criminology, Faculty of Law, VU University Amsterdam, Amsterdam, 1081HV, Netherlands; 3 Netherlands Institute for the Study of Crime and Law Enforcement, Amsterdam, 1081HV, Netherlands; 4 Faculty of Philosophy, VU University Amsterdam, Amsterdam, 1081HV, Netherlands; 5 Tilburg Law School, Tilburg University, Tilburg, 5037DE, Netherlands; 6 Department of Clinical Psychology, VU University Amsterdam, Amsterdam, 1081BT, Netherlands; 7 EMGO Institute for Health and Care Research, VU University and VU University Medical Center Amsterdam, Amsterdam, 1081BT, Netherlands; 8 Leuphana University, Lüneburg, D-21335, Germany

**Keywords:** prison, offenders, executive functions, CANTAB, running therapy, physical activity, Actiwatch, sleep

## Abstract

**Background: **Executive dysfunction appears to be related to increased recidivism. Of note is that sleep disturbances, which are highly prevalent in prisons, may attenuate executive functions. Thus, improving executive functions, either directly or indirectly through the improvement of sleep, may reduce recidivism. It is hypothesised that physical exercise, in the form of Running Therapy, has a direct positive effect on executive functions as well as an indirect effect through the improvement of sleep.

**Methods/Design: **Seventy two (N = 72) detainees in various penitentiary institutions in the Netherlands will be recruited in this study. A baseline measurement, including six neuropsychological tests of the Cambridge Neuropsychological Test Automated Battery (CANTAB), an assessment of sleep quality and duration using the Actiwatch (Actiwatch 2, Philips Respironics, Murrysville, PA, USA) and various other measurements will be administered before the start of the treatment. After 3 months of Running Therapy, participants will be assessed again with the same tests for neuropsychological and physical functioning. Primary outcomes are executive functioning and various sleep variables.

**Discussion: **This study will be the first to investigate the possible influence of Running Therapy on the cognitive functioning, sleep and aggression in prisoners.

## Background

International studies report that 35 to 67 percent of released prisoners are detained for reoffending within approximately 2 to 3 years ^1– 4^. These percentages show that the reduction of recidivism is of great importance to society, since crime carries a great (financial) burden ^5^.

The risk of reoffending appears to be negatively related to executive functioning ^6– 8^. Executive functions are higher order cognitive functions including planning, working memory, taking initiatives, set-shifting, attention, and impulse control ^9, 10^, and are crucial for self-regulation ^11^. Planning and goal-directed behaviour are essential for successful re-entry into society, as ex-prisoners face complex challenges such as finding housing and employment ^12^. Another important executive function is impulse control, which enables us to regulate and suppress aggressive behaviour, for example ^9^; reduced impulse control thus increases the risk of aggressive behaviour.

Executive functions may be improved by physical activity ^13, 14^. For example, brisk walking was found to improve impulse control in older adults ^14^. An increase in impulse control after participation in a physical activity programme is quite a consistent finding ^13, 15, 16^ and appears especially effective in sedentary people ^13^. Prison life consists mostly of passive leisure activities such as watching television ^17^, and physical inactivity is a hallmark of prison life in various countries ^18– 21^. So, a large percentage of the prison population has a sedentary lifestyle.

Closely related to both executive functioning and physical (in)activity is sleep. Sleep disturbances may diminish executive functioning ^22^, while increased physical activity may improve sleep ^23, 24^. Sleep disturbances are highly common in prison and are responsible for a relatively large part of prison health care use ^25, 26^.

Besides sleep disturbances, various psychiatric disorders are also highly prevalent in prisons, such as depression ^27^, anxiety disorders and ADHD ^28– 30^. These psychiatric disorders may also negatively affect sleep ^31^. Exercise, e.g. Running Therapy, may indeed reduce symptoms of depression and anxiety ^32^ and behavioural symptoms of ADHD ^33, 34^.

In sum, physical activity may positively influence executive functions directly, but also indirectly, through improved sleep. We therefore hypothesize that physical exercise, in the form of supervised Running Therapy, will improve executive functioning and sleep, and will reduce aggression in prisoners. Although a randomized controlled trial (RCT) would be the best option to test this hypothesis, our current study could be considered a pilot behavioural intervention trial. Conducting an RCT directly is not possible, as the participating prisons were currently not willing to withhold treatment for some prisoners (i.e. the control group). However, this pilot study may eventually lead to an opportunity to conduct an RCT.

## Methods and analysis

### Study design

This study concerns a prospective cohort study, measuring neuropsychological performance and sleep of detainees before and after receiving 3 months of Running Therapy. No further experimental intervention will take place, and this study does not interfere with or alter daily programs, treatments or any other environmental factors.

### Participants

Prisoners (male adults) of two Penitentiary Institutions in the Netherlands (i.e. PI Leeuwarden and PI Ter Apel) that are referred to Running Therapy by the psycho-medical staff will be recruited in this study. Various complaints or disorders may result in referral to Running Therapy, e.g. ADHD, sleep disturbances, anxiety disorders and depression. All participants in Running Therapy who speak sufficient Dutch or English will be approached and asked to participate in this study. In addition, we will attempt to approach those who do not speak Dutch or English by using a translation phone service often used in Penitentiary Institutions by the psycho-medical staff. As we are not allowed to interfere with daily practice in prison, participants will continue to receive treatment-as-usual from the prison care, which may entail changes in drug regimen or participation in other interventions. Participants may be excluded from Running Therapy when they sustain an injury or when they behave aggressively. Such decisions are made by the running therapist in concordance with the prison staff.

### Intervention

Running therapy is already part of the regular care in the Penitentiary Institutions where this study will be conducted. Prisoners are referred to Running Therapy by the psycho-medical staff, which consists of the psychologist, psychiatrist, physician and nursing staff. Of note is that this study does not make any modifications to the Running Therapy, and studies the intervention as is.

***Running Therapy.*** This aerobic exercise group-therapy consists of supervised running for 2 days per week and 1 day per week of unsupervised running. During the therapy, the therapist encourages participants to run at a moderate pace and keeps track of the goals and sub-goals of the participants. The main goal of the therapy is to be able to run consecutively for 30 minutes at a moderate pace, three times per week, which will be achieved after 15 weeks of Running Therapy (see Appendix A for the complete program). Of note is that the therapy does not solely consist of running, but also contains a social aspect (running in a group, talking with participants or the therapist) and a reward aspect (achieving sub goals).

### Setting

The Running Therapy is currently offered in two Penitentiary Institutions in the Netherlands, PI Ter Apel and PI Leeuwarden, and the study will take place in both these institutions.

### Measurements and procedures

Participants will undergo two measurements: a baseline measurement before starting with Running Therapy and a post measurement after 3 months of Running Therapy. The baseline measurement takes place over a single day and will take approximately 90 minutes in total. At the baseline measurement, anamnesis takes place and the participant will be assessed with a neuropsychological test battery, the Cambridge Neuropsychological Test Automated Battery (CANTAB). In addition, participants are given four questionnaires (see “Secondary Outcome Measures” for more information) to fill out in their own time, and are instructed to bring these with them to the first day of Running Therapy. Optionally, an Actiwatch (Actiwatch 2, Philips Respironics, Murrysville, PA, USA) will be handed to the participant, which will be worn for 7 consecutive days (for more information about the Actiwatch, refer to “Primary outcome measures – Sleep”.

After three months of participating in Running Therapy, the participant will undergo the same tests as taken when assessing the baseline measurement. The four questionnaires and the Actiwatch will again be handed out to the participant.

According to the policy of the Dutch Custodial Institutions Agency (DJI), we are not allowed to provide the participant with an incentive. However, the measurements will be planned during the moments in which participants are usually spending time in their cell. We have experienced that prisoners regard the measurements as a welcome change.

### Primary outcome measures

***Executive functions***

The CANTAB ^35^ is a computerised neuropsychological test battery and is used to assess the executive functions of the participants. A 12.1 inch touchscreen tablet is used to administer the test. In two tests, a two-buttoned press pad is used. Few studies have assessed the reliability and validity of the CANTAB. One older study, that included some, but not all tests included in the current study, showed that test-retest correlations are above 0.6 for most of the subtests ^36^. The main reason that we chose this battery is a practical one; neuropsychologically testing a prisoner can sometimes be logistically challenging. Using this highly portable test battery, that can be used in any room with a table and two chairs, provides us with the necessary flexibility to test participants anywhere the prison staff wants us to.

The following six CANTAB tests will be administered.

*SOC.* Stockings of Cambridge measures planning, and is similar to the commonly used Tower of London (TOL). The main outcome measure is the number of problems solved in the minimum number of moves.

*SWM.* Spatial Working Memory measures the ability to retain and manipulate information in the working memory and heuristic strategy. The main outcome measure is total number of errors made.

*SST.* The Stop Signal Task measures response inhibition. The main outcome measure is SSRT, which is calculated by subtracting the mean stop-signal delay (the time between the stimulus and the beep) from the median go reaction time (the median response time on trials without a beep).

*IED.* Intra-Extra Dimensional Set-Shift measures set-shifting and is similar to the commonly used Wisconsin Card Sorting Test. The main outcome measure is the total number of errors (adjusted for the number of trials conducted).

*CRT.* Choice Reaction Time is a simple reaction time assessment and is used to measure reaction time and attention. The main outcome measures are mean reaction time, and the difference in mean reaction time between the second and the first half.

*CGT.* The Cambridge Gambling Task measures decision-making impulsivity and risk-taking behaviour. The main outcome variables are measures of risk-taking, risk-adjustment, quality of decision making, delay aversion and overall proportion bet.

***Sleep***

*Actiwatch.* The Actiwatch AW2 (Respironics, Philips) will be used to measure the rest-activity rhythm, specifically sleep. Actiwatches are small activity monitors that are worn on the wrist, like a wristwatch, 24 hours a day. The Actiwatch gathers data by measuring the amount and intensity of movements made within every 5 seconds (which is the chosen epoch length). The Actiwatch stores the movement data of each epoch separately, which is retrieved later using a reader connected to a PC. The device's wristwatch-like design reduces physical discomfort to a minimum.

The Actiwatch provides information on the (in)stability of the rest-activity rhythm from one day to another (Inter-daily Stability; IS), and on the fragmentation of the rest-activity rhythm within the day, i.e. changes from periods of rest to activity and vice versa (Intra-daily Variability; IV). It also indicates the difference between maximal activity and maximal rest (Relative Amplitude; RA), and provides data on the 10 most active hours (M10) and the 5 least active hours (L5; for detailed information about these measurements, see the following references ^37, 38^).

Sleep analysis software (Philips Actiware 6.0.4, Respironics Inc.) will be used in order to analyse sleep. Sleep analysis will produce variables such as time spent in bed, sleep efficiency, sleep onset latency and total sleep duration.

*Sleep-wake diary.* Sleep-wake diaries are used to assess several events/activities such as time of medication use, use of caffeine/nicotine, bed time, wake time and time spent exercising. The sleep-wake diary is mainly used to improve the accuracy of sleep analysis.

### Secondary outcome measures

*SCL-90-R.* The Symptom Checklist-90-Revised ^39^ is used to assess a broad spectrum of complaints, such as pain, depression and hopelessness, and with the SCL-90-R, we are able to reliably assess clinically significant change ^40^. Test-retest correlations, for a 10-week interval, were found to range from r = .68 to .80 ^41^.

*SDL.* The Sleep Diagnosis List ^42^ is a self-report questionnaire that consists of 75 statements related to sleep and symptoms of sleep disorders. It will be used as a subjective measure of sleep and to control for disorders associated with sleep disturbances. The SDL is based on the Sleep Diagnosis Questionnaire ^43, 44^ and has been validated in a large Dutch population with sleep disorders ^45^.

*PSQI.* The Pittsburgh Sleep Quality Index ^46^ is a commonly used self-report questionnaire and is used to assess e.g. sleep quality and sleep duration. Internal consistency of the PSQI ranges from 0.70 to 0.80 (Cronbach’s alpha) and the PSQI is known to have a good construct validity ^47^; PSQI scores are more highly correlated to sleep disturbances (r = .69 to .77) than to e.g. mood and depression (r = .22 to .65). In patients with primary insomnia, the PSQI has been shown to have a high test-retest reliability, with r = 0.87 ^48^.

*AVL.* The Aggression Questionnaire ^49^ is a self-report questionnaire that consists of 29 statements that are related to aggressive thoughts and behaviour. While internal consistency for the global aggression scale and three subcomponents are sufficient (Cronbach’s alpha > 0.7), the internal consistency of the verbal aggression subcomponent is insufficient (Cronbach’s alpha = 0.5). Test-retest correlations for all subcomponents and the total score are high, r > 0.76.

*Activities.* Participants are asked to provide an estimate of their participation in activities such as sports, labour and outdoor time on a 4-point Likert scale (never, sometimes, often, always). In addition, participants are asked to estimate the average hours per day spent on activities such as watching television and reading.

*Demographic and control variables.* At baseline, demographic variables such as age, level of education, type of crime, number of previous incarcerations and current medication use are gathered.

### Statistical analysis

For the main research questions (i.e. does running therapy improve cognition and sleep?), a repeated measures AN(C)OVA will be conducted for the results on the CANTAB and for the various sleep variables, comparing the baseline results with the post-measurement results. The main analyses will be conducted according to the intention-to-treat principle. Missing data will be imputed using the multiple imputation function as provided in SPSS 21 (IBM Corp, Armonk, NY, USA).

### Sample size calculation

A sample size calculation was made using G*Power version 3.1.3 ^50^. Since no similar studies have been conducted, the effect size was set small-to-moderate ( *f* = 0.15). The lowest test-retest correlation of one of the used CANTAB tests was used as input for the correlation between repeated measures (r = 0.6).

In sum, effect size was set to *f* = 0.15, with an alpha error probability of *p* = 0.05, power of 1-β = 0.8, 1 group, 2 moments of measurement and correlation among repeated measures of r = 0.6. In G*Power, this resulted in a total sample size of N = 72.

### Ethical and legal considerations

This study protocol has been reviewed by CERCO (Committee of Ethics in studies of Law and Criminology), the ethical committee of the faculty of Law at VU University, which declared it saw no objections to the study. In addition, the accredited medical ethical committee of the VU medical centre provided an official declaration (reference number 2014.399) that this study does not need further medical ethical approval, because of the low burden and non-medical non-interventional nature of the study (i.e. the intervention is already part of the institutional care and therefore requires no additional ethical approval). This study has been submitted for registration in the Dutch Trial Register (Nederlands Trial Register); the identification number will be made available in a revised version of this article.

Information letters and Informed Consent forms will be translated in various languages, to ensure that participants can read them in their own language. Data will be coded using a chronological number in combination with an identifier for the institution; the first participant in PI Ter Apel, for example, will be coded as “TA_001”. All non-anonymous data, such as the Informed Consent forms will be stored in the Penitentiary Institutions, as required by the regulations of the Custodial Institutions Agency (DJI). Anonymous data will be stored and analysed at the VU University Amsterdam. Any researcher who works with the participants (i.e. any researcher who could violate the privacy of the participants) is obliged to sign a confidentiality agreement, as required by the regulations of DJI. Students of VU University who will work on this dataset, for example for a thesis project, are required to sign a confidentiality agreement provided by VU University.

### Access to data

The researchers of this study, as well as their students working on this project, will have unlimited access to the dataset. The dataset will be made available to colleagues and peer-reviewers upon request; official approval of DJI may be required beforehand. Variables containing sensitive information may be removed before sharing the dataset, such as date of birth. Participants will not be allowed to see the final data.

### Dissemination

This study will be finished in the second half of 2016, and the results will be published in international peer reviewed scientific journals.

## Discussion

The main purpose of this study will be to investigate the influence of Running Therapy on executive functioning, sleep and aggression of prisoners. To our knowledge, this will be the first study of its kind in a prison population, which may provide results that are relevant to prison administrations, policymakers, and prison clinicians.

Different aspects of Running Therapy may have a positive influence on the prisoner population. Firstly, regular exercise may positively influence executive functions, in particular impulse control ^13, 14^, which could be of importance in reducing recidivism. Secondly, acute bouts of exercise may also improve executive functions such as attention, memory, reasoning and planning ^15, 51^, also making Running Therapy of added value in the short term. For example, it may be useful to plan Running Therapy sessions right before other activities or therapy sessions to improve attentional performance and increase participation of the prisoners. Furthermore, although investigating the influence of Running Therapy on depressive symptoms is a secondary objective of the current study, Running Therapy may have significant clinical impact on these symptoms as well. In general, aerobic exercise has a small-to-moderate effect on depressive symptoms ^32, 52^, which could improve cognition, sleep, and general well-being.

As antisocial personality disorder is highly prevalent in prison (65%) ^53^, this study may also be the first to examine the effects of exercise on the cognition of people with antisocial personality disorder. As impulsivity and/or failure to plan ahead is a clinical hallmark of these patients ^54^, improving executive functions such as impulse control or planning may be of added value in treatment.

Studying an intervention such as Running Therapy in prison comes with challenges and considerable limitations. One such limitation is that it we will be conducting a Phase I/II study instead of an RCT, as the participating prisons were not ready to directly allow an RCT. A follow-up RCT would be needed to confirm any positive results. Another limitation is that rewards for participation are not allowed, making it more challenging for us to recruit participants. An example of a logistic issue is the difficulty in planning a meeting with a participant with sufficient time to administer all the tests and questionnaires. Our solution is to ask participants to fill out a number of the questionnaires in their own time and bring these with them to therapy a week later, which might result in a somewhat lower compliance rate for these questionnaires. Another limitation concerns the use of actigraphy to measure sleep. Polysomnography is considered the gold standard in sleep research, with actigraphy being the second most valid method. However, as it not within our possibilities to temporarily transport all the participants to a sleep research lab for polysomnographic research, for this study, we consider actigraphy to be the most suitable and valid method; a clear advantage of actigraphy is the possibility to study the participants in the environment they reside in. One limitation of actigraphy is that we know from previous experience in prison, a number of the participants choose not to wear the Actiwatch, as they fear it might affect their social status with their fellow inmates. These limitations are examples of the challenges one faces when conducting research in a prison environment. In our view, however, prisoners are an important population with great impact on society, and researchers should not be discouraged to conduct research in this setting. Instead, we should try to build expertise conducting research in this particular environment, and hopefully, the results of our study will help to pave the way to the possibility to conduct RCTs in prison in the future.

In sum, if the results are indeed in accordance with our hypotheses, Running Therapy may eventually prove to be a useful approach to improve executive function, and possibly reduce aggressive behaviour and psychiatric symptoms of depression, ADHD, sleep disturbances and anxiety in prisoners.
