# Supplementary material for: Study Protocol: The influence of Running Therapy on executive functions and sleep of prisoners
Source: F1000Res. 2015 Jun 15;4:152. [Version 1] doi: 10.12688/f1000research.6469.1 (PMC4654434; doi:10.12688/f1000research.6469.1)
Supplement: Peer review history

# Peer review: Referee response for version 1

**DOI:** 10.5256/f1000research.6941.r11146

**Published:** 2015-11-17

**Authors:** Thomas Kleinsorge, Viktoriya Maydych

**Affiliations:** 1 Leibniz Research Centre for Working Environment and Human Factors (IfADo) , Dortmund, Germany

The authors present a protocol for a study-in-preparation that aims to investigate effects of Running Therapy on executive functions and sleep quality in prisoners. Study participants in two Dutch prisons will receive three months of Running Therapy consisting of supervised running for 2 days per week and 1 day per week of unsupervised running. Before starting with Running Therapy the prisoners will be assessed with a neuropsychological test battery examining executive functions (CANTAB) and will be given four questionnaires (and optionally an Actiwatch) measuring a broad spectrum of complaints, sleep characteristics and aggression. After three months of participating in Running Therapy, the participants will be re-assessed with the same tests and questionnaires. The application of Running Therapy is supposed to positively influence executive functioning, sleep and to reduce aggression as well as symptoms of depression, anxiety and behavioural symptoms of ADHD.

General evaluation

The authors propose an innovative study that tackles a highly relevant applied issue, namely to identify measures that possibly reduce recidivism among prisoners. As the authors are aware, this relevance in practical terms goes along with some limitations in terms of meeting scientific standards. These limitations are imposed by severe restrictions regarding the study design as well as the examination procedures. Because the investigators are not allowed to interfere with any daily practice in prison, the authors are obliged to resort to a pre-post design instead of conducting a randomized controlled trial. Furthermore, the authors are not allowed to control the referral of potential participants to Running Therapy but will include any participants referred to this therapy by the psycho-medical staff of the respective institution. Despite these limitations, the proposed study promises to yield important data that will hopefully pave the way to a more rigorous study of this issue.

Specific comments

 

1. One of the major problems associated with a pre-post design is that practice effects may be erroneously considered as treatment effects. Would it be possible to run a control group of not imprisoned subjects that is matched with respect to other relevant characteristics in order to estimate the magnitude of to-be expected practice effects?
2. The authors discuss both direct and indirect routes by which Running Therapy may improve executive functions. Maybe it would be possible to distinguish among possible modes of action by running mediational analyses?
3. The measures obtained with Actiwatch offer the opportunity to analyze effects of Running Therapy on sleep quality and other activity related measures in a temporally more fine-grained manner than the primary outcome measures related to executive functions, for example, by using multi-level analyses. Overall, given that prisons are a very hard to access field of observation, it should be worthwhile to make use of more than standard statistical procedures to gather as much information as possible from any data obtained in this setting.

We have read this submission. We believe that we have an appropriate level of expertise to confirm that it is of an acceptable scientific standard.

# Peer review: Referee response for version 1

**DOI:** 10.5256/f1000research.6941.r11147

**Published:** 2015-11-13

**Authors:** Joseph HR Maes

**Affiliations:** 1 Donders Institute for Brain, Cognition and Behavior, Radboud University Nijmegen, Nijmegen, Netherlands

This research proposal describes the details of a pilot study to assess the effect of a running therapy on the quality of sleep and executive functioning (EF) in prison inmates. The plan is based on previous literature suggesting: 1) a negative relationship between executive functioning and aggression and chance of recidivism in offenders; 2) a positive relationship between physical activity and EF; 3) a positive association between physical activity and sleep quality; 4) a negative association between sleep disturbances and EF; 5) a positive association between imprisonment and sleep disturbances. Given that prison life is characterized by physical inactivity, the points 1‒5 suggest that  a running therapy may improve EF  either directly or indirectly by improving sleep quality. The improvement in EF in turn might imply a decrease in aggression and recidivism rate. The idea is to have the research embedded within ongoing practices in two penitentiary institutions in the Netherlands. Specifically, the study is proposed to be performed in prisoners that are referred to a running therapy by the psycho-medical staff of the institutions as part of regular treatment interventions. Basically, the main study involves performing pre- and post-running therapy measurements using a variety of instruments to assess aspects of EF and sleep.

The basic idea behind the proposal seems to logically follow from the existing pieces of knowledge concerning the interrelationships between imprisonment,  aggression, recidivism, physical activity, EF, and sleep. Moreover, the proposal is well-written and carefully addresses practical,  ethical, and legal considerations, and may set the stage for future, more controlled research within the prison context. For these reasons, I approve the manuscript.

However, the practical limitation of not being able to perform a randomized controlled trial automatically implicates the major methodological weakness of the proposed study. That is, the absence of a control group, not receiving the target intervention, prevents us from determining  whether any differences  found in EF indices between the two measurements are due to the running therapy as such or to a general  practice or loss-of-novelty effect, or test-retest unreliability (measurement error). The various measures from the CANTAB may differ in their susceptibility to the latter type of non-treatment-related effects. For example, especially the IED test from the CANTAB may be particularly vulnerable for practice effects (participants may have become aware of the ‘trick’ of shifting relevant stimulus dimensions). Also with respect to potential changes in sleep parameters, the absence of a control group prevents us from unequivocally associate them with a running therapy effect. Given this weakness, the authors could try to get the most out of their data by considering the following methodological and analytical points.

1. From the description under Participants, I understand that not all prisoners are eligible to take part in the running therapy. Would there be any objections by the participating prisons to ask to have these individuals also perform the EF tests, wearing the Actiwatch, and filling out the questionnaires? In this way, these participants could serve as a control group, being helpful in interpreting the cause of any changes observed in EF or sleep parameters in the ‘experimental’/running therapy group. Of course, such ‘control group’ would be far from ideal, probably not being matched on many relevant characteristics and potentially showing large baseline differences with the treatment group, but it would be better than not having a control group at all.
2. There are various ways to try to diminish the influence of practice and test-retest reliability issues. Although each of the available techniques has its own problems and limitations, one could for example use  the technique of dual basement assessment to decrease the influence of practice effects. Alternatively, one could use statistical means to try to minimize their influence (e.g., see Collie *et al.*, 2002).
3. The proposed statistical analysis does not (explicitly) address the issue of sleep mediating a possible effect of the running therapy on (parameters of) EF. Perhaps one could try to perform a formal mediation analysis, in which some index of the success of the running training (e.g., difference  in mean speed or distance run in a given period of time at baseline and at the end of training) as predictor, some (compound) index of change in sleep quality as mediator, and some (compound) measure of EF improvement as criterion. In this way, one could also get (preliminary) information on causal relationships. For example, it could, at least theoretically, also be the case that sleep problems are caused by poor EF (e.g., not being able to inhibit rumination causes sleep problems), suggesting that improved EF mediates the association between the effect of the running therapy on sleep rather than the other way around.

I have read this submission. I believe that I have an appropriate level of expertise to confirm that it is of an acceptable scientific standard.
